# *LINC00240* in the 6p22.1 risk locus promotes gastric cancer progression through USP10-mediated DDX21 stabilization

**DOI:** 10.1186/s13046-023-02654-9

**Published:** 2023-04-18

**Authors:** Nasha Zhang, Bowen Wang, Chi Ma, Jiajia Zeng, Teng Wang, Linyu Han, Ming Yang

**Affiliations:** 1 Department of Radiation Oncology, Shandong Cancer Hospital and Institute, Shandong First Medical University and Shandong Academy of Medical Sciences, Shandong Province, Jinan, 250117 China; 2 Shandong Provincial Key Laboratory of Radiation Oncology, Cancer Research Center, Shandong Cancer Hospital and Institute, Shandong First Medical University and Shandong Academy of Medical Sciences, Shandong Province, Jinan, 250117 China; 3 Jiangsu Key Lab of Cancer Biomarkers, Prevention and Treatment, Collaborative Innovation Center for Cancer Personalized Medicine, Nanjing Medical University, Nanjing, 211166 Jiangsu Province China; 4 Department of Thyroid Surgery, Affiliated Yantai Yuhuangding Hospital of Qingdao University, Shandong Province, Yantai, 264000 China

**Keywords:** LINC00240, DDX21, USP10, Gastric cancer, Ubiquitination

## Abstract

**Background:**

Gastric cancer remains the leading cause of cancer death in the world. It is increasingly evident that long non-coding RNAs (lncRNAs) transcribed from the genome-wide association studies (GWAS)-identified gastric cancer risk loci act as a key mode of cancer development and disease progression. However, the biological significance of lncRNAs at most cancer risk loci remain poorly understood.

**Methods:**

The biological functions of *LINC00240* in gastric cancer were investigated through a series of biochemical assays. Clinical implications of *LINC00240* were examined in tissues from gastric cancer patients.

**Results:**

In the present study, we identified *LINC00240*, which is transcribed from the 6p22.1 gastric cancer risk locus, functioning as a novel oncogene. *LINC00240* exhibits the noticeably higher expression in gastric cancer specimens compared with normal tissues and its high expression levels are associated with worse survival of patients. Consistently, *LINC00240* promotes malignant proliferation, migration and metastasis of gastric cancer cells in vitro and in vivo. Importantly, LINC00240 could interact and stabilize oncoprotein DDX21 via eliminating its ubiquitination by its novel deubiquitinating enzyme USP10, which, thereby, promote gastric cancer progression.

**Conclusions:**

Taken together, our data uncovered a new paradigm on how lncRNAs control protein deubiquitylation via intensifying interactions between the target protein and its deubiquitinase. These findings highlight the potentials of lncRNAs as innovative therapeutic targets and thus lay the ground work for clinical translation.

**Supplementary Information:**

The online version contains supplementary material available at 10.1186/s13046-023-02654-9.

## Introduction

Gastric cancer remains the leading cause of cancer death in the world, with an estimated 769,000 deaths (7.7% of the total cancer deaths) according to the GLOBOCAN estimates in 2020 [1]. Incidence rates in Eastern Asia and Eastern Europe are generally high, whereas rates are low in Northern America and Northern Europe [1]. Established environmental risk factors of gastric cancer include *Helicobacter pylori* (*H. pylori*), alcohol consumption, tobacco smoking, foods preserved by salting, and low fruit intakes [1, 2]. Chronic *H. pylori* infection is the main cause of gastric cancer [1, 2]. The prevalence of *H. pylori* infection is extremely high, infecting about half of the world's population [1, 3]. However, only less than 5% of infected hosts will develop gastric cancer, indicating that other factors, especially differences in host genetics, may be crucial during tumorigenesis of stomach.

Multiple genetic loci have been significantly associated with gastric cancer risk via genome-wide association studies (GWASs) [4–14]. However, only a few of gastric cancer-susceptibility genes in these risk loci have been functionally verified [4, 6, 9, 13, 15]. Multiple studies elucidated that long noncoding RNAs (lncRNAs) are transcribed from cancer risk genetic loci and contribute to tumorigenesis [15–21]. For instance, we previously reported that *lncPSCA* in 8q24.3 is a novel tumor suppressive gene [15]. *LncPSCA* remarkably inhibits malignant behaviors of gastric cancer cells in vitro and in vivo. Oncoprotein DDX5 has been identified as the interacting protein of lncPSCA. Interestingly, lncPSCA could accelerate degradation of DDX5 via the ubiquitin–proteasome pathway. Decreased levels of DDX5 results in less RNA polymerase II (Pol II) binding with DDX5 as well as transcriptional activation of multiple P53 signaling tumor suppressors by Pol II [15]. However, the role of lncRNAs transcribed from genome intervals around other gastric cancer risk signals in development and progression of gastric cancer are still largely unclear.

In the current study, we systematically investigated three candidate lncRNAs (*LINC00240*, *LINC01012* and *ZNRD1-AS1*) transcribed from the 6p22.1 risk locus. Only *LINC00240* exhibits the noticeably higher expression in gastric cancer specimens compared with normal tissues and its high expression levels are associated with worse survival of gastric cancer patients. Consistently, *LINC00240* promotes malignant proliferation, migration and metastasis of gastric cancer cells in vitro and in vivo. Importantly, LINC00240 could interact and stabilize oncoprotein DDX21 via eliminating its ubiquitination by its novel deubiquitinating enzyme USP10, which, thereby, promote progression of gastric cancer.

## Material and methods

### Patients and tissue specimens

There are two gastric cancer patient cohorts (Discovery cohort and Validation cohort) which were enrolled in the current study. The detailed characteristics of all patients of Discovery cohort (*n* = 96) or Validation cohort (*n* = 30) from have been described previously. This study was approved by the Institutional Review Board of Shandong Cancer Hospital and Institute. At recruitment, the written informed consent was obtained from each subject. The methods were carried out in accordance with the approved guidelines.

### Quantitative reverse transcription PCR (RT-qPCR)

Total RNA was isolated from tissue specimens or culture cells with Trizol reagent (Invitrogen, USA) as previously reported [15, 22]. Each RNA sample was then reverse transcribed into cDNAs using PrimeScript™ RT Master Mix (TaKaRa, Japan). cDNA and appropriate primers (Supplementary Table 1) were plated in a 96-well plate and gene expression levels were measured using TB Green Premix Ex Taq II (TaKaRa, RR820A). The expression of candidate lncRNAs or genes was calculated by using the 2^−ΔΔCt^ method. Each sample was measured in triplicate and specificity of PCR products was validated by the melting-curve.

### Cell culture

Human gastric cancer MKN-45, MKN-28, and BGC-823 cells were cultured in RPMI 1640 medium (Gibco, USA). Human HGC-27, GES-1, MGC80-3 and HEK293T cells were cultured in DMEM medium (Gibco, USA). Human gastric cancer AGS cells were cultured in Ham's F-12 K medium (Gibco, USA). HGC-27, MGC80-3, MKN-45, and HEK293T cells were kindly provided by Dr. Yunshan Wang (Jinan Central Hospital, Shandong Province, China). GES-1, MKN-28, AGS, and BGC-823 cells were kindly provided by Dr. Jie chai (Shandong Cancer Hospital and Institute, Shandong Province, China). All media were supplemented with 10% fetal bovine serum (FBS) (Gibco, USA). Cells were maintained at 37 °C in a 5% CO_2_ incubator and periodically tested mycoplasma negative.

### The expression and shRNA constructs and transient transfection

The human full-length *LINC00240* (NR_026775.2) cDNA and truncated versions of *LINC00240* cDNA were directly synthesized and cloned after the CMV promoter of pCDH-CMV-MCS-EF1-Puro. The full-length *LINC00240* cDNA plasmid was named as LINC00240. After one HA-tag sequence was inserted after ATG of the CDS region of *DDX21* (NM_004728.4), the cDNA was cloned into the pcDNA3.1 vector to generate the HA-tagged DDX21 plasmid (WT). Five truncated DDX21 plasmids (ΔDEADc, Δ1-398, Δ617-783, ΔGUCT, and ΔAdoMet) were mutants of the HA-tagged DDX21 plasmid with CDS region after deletion of the DEADc domain, 1-398aa, 329-1283aa, the GUCT domain, or the AdoMet domain, separately. Two shRNA hairpins targeting human *LINC00240* (sh240-1 or sh240-2) or the control shRNA (Supplementary Table 2) were cloned into the pLKO.1 vector. The resultant plasmids were designated sh240-1, sh240-2, or shNC. All the plasmids were synthesized by Genewiz (Suzhou, China) and sequenced to confirm the orientation and integrity. Transient transfection of plasmid constructs or small interfering RNAs (GenePharma, Shanghai, China) (Supplementary Table 2) was performed with Lipofectamine 3000 or the INTERFERin reagent (Polyplus, 409–10).

### Lentiviral transduction

As previously reported, recombinant lentiviral particles were produced by transient co-transfection of the sh240-1, sh240-2, or LINC00240 plasmids into HEK293T cells [23]. After transfection for 48 h and 72 h, the viral supernatants containing the recombinant lentiviral particles of LINC00240, sh240-1, sh240-2 or their controls (NC or shNC) were collected and filtered. Gastric cancer HGC-27 and MGC80-3 cells were infected with the viral supernatants containing 8 μg/mL polybrene. Stably *LINC00240-*overexpression (OE) cells were selected using 2 μg/mL puromycin. Stably *LINC00240*-knockdown (KD) gastric cancer cells were selected using 10 μg/mL blasticidin. In these lentiviral transducted cells, the expression levels of *LINC00240* were examined by RT-qPCR.

### Cell proliferation and colony formation analyses

For cell proliferation assays, a total of 3 × 10^4^ gastric cancer HGC-27 or MGC80-3 cells with stable overexpression of *LINC00240* or silencing of *LINC00240* were seeded in 12-well plates. Cells were harvested and counted at 24 h, 48 h, and 72 h after seeding. For the rescue assays, siRNAs of *DDX21* or NC RNA (Genepharma, China) (Supplementary Table 2) were transfected to the stably *LINC00240-*OE HGC-27 or MGC80-3 cells, whereas the HA-tag *DDX21* expression construct or the pcDNA3.1 vector were transfected to the stably *LINC00240-*KD cells. For colony formation assays, a total of 1,000 gastric cancer cells per well were seeded in 6-well plates. When colonies were visible after 14 days, cells were washed with cold PBS twice and fixed with the fixation fluid (methanol:acetic acid = 3:1). After the gastric cancer cells were dyed using crystal violet, colony number in each well was counted.

### Xenograft studies

To examine the in vivo role of *LINC00240*, we inoculated subcutaneously a total of 6 × 10^6^ stably *LINC00240-*OE, *LINC00240*-KD or control HGC-27 cells into fossa axillaries of five-week-old female nude BALB/c mice (Vital River Laboratory, Beijing, China). Tumor growth was measured every two days as previously described [15, 23]. To assess functions of *LINC00240* during gastric cancer metastases in vivo, we injected a total of 2 × 10^6^ HGC-27 cells with stable *LINC00240*-KD (shNC, sh240-1, or sh240-2) or *LINC00240*-OE (NC or LINC00240) into tail vein of 5-week-old female nude BALB/c mice (Vital River Laboratory, Beijing, China) (*n* = 3 per group). Mouse lungs with metastatic tumors were formalin fixed, paraffin-embedded and stained with hematoxylin and eosin (HE). Immunohistochemistry (IHC) staining was performed in mouse livers with an antibody specific for vimentin as previously reported [24, 25]. All procedures involving mice were approved by the Animal Care Committee of Shandong Cancer Hospital and Institute. All analyses were performed in a blinded fashion with individuals unaware of xenograft types.

### Wound healing and transwell assays

In wound healing assays, a wound was scratched by a 10 μL pipette tip when the cell layer of gastric cancer cells with lentiviral transduction of overexpressed *LINC00240* or the *LINC00240* shRNAs reached about 90% confluence. Cells were continued cultured at 37 °C with 5% CO_2_ and the average extent of wound closure was quantified. For transwell assays, gastric cancer cells were added to upper transwell chambers (pore 8 μm, Corning) after transwell chambers were coated with BD Biosciences Matrigel (1:20 dilution) for 12 h. Culture medium containing 10% FBS was added to the lower wells. After 48 h, gastric cancer cells migrated to the lower wells through pores were stained with 0.2% crystal violet solution and counted.

### Subcellular fractionation

According to the manufacturer’s instructions, the cytosolic and nuclear fractions of gastric cancer HGC-27 or MGC80-3 cells were separately isolated using the nuclear/cytoplasmic Isolation Kit (Biovision, K266, China). The relative RNA levels of LINC00240 in cytosolic or nuclear fractions were detected by RT-qPCR.

### RNA pulldown

The RNA pulldown assays were performed to identify proteins interacting with LINC00240 as reported previously [15, 23]. To prepare the DNA template for in vitro RNA synthesis, *LINC00240* was subcloned into pcDNA3.1 with inserted T7 promoter before and after the cloning site. In briefs, LINC00240 RNAs were transcribed with T7 RNA polymerase (MEGAscript T7 Transcript Kit, Thermo fisher, AM1330) and the linearized *LINC00240* construct. After purified with the RNeasy minikit (Qiagen, #74,104, Germany), the sense and antisense LINC00240 RNAs were biotinylated with Pierce™ RNA 3' End Desthiobiotinylation Kit (Thermo fisher, 20,163). These RNAs were then incubated with MGC80-3 protein extracts at 4 °C for 1 h. Proteins bound on the streptavidin magnetic beads were recovered with Elution Buffer following the instruction of Pierce™ Magnetic RNA–Protein Pull-Down Kit (Thermo, 20,164, USA). The retrieved proteins were then analyzed by liquid chromatography-tandem mass spectrometry (LS-MS/MS) (Hoogen Biotech Co., Shanghai, China) and Western Blot. Mass spectra were analyzed using MaxQuant software (version 1.5.3.30) with the UniProtKB human database (Uniport *Homo sapiens* 188441_20200326).

### RNA immunoprecipitation (RNA-IP)

As reported previously, RNA-IP assays were performed with the DDX21 antibody or the IgG Isotype-control [15, 23]. The protein-RNA complexes were recovered by Dynabeads® Protein G beads (Thermo, 10003D, USA). LINC00240 RNA levels in the precipitates were measured by RT-qPCR. A total of 10% of inputs were used for RT-qPCR.

### Western blot

Western blot was performed following the standard protocol as previously described [15, 23]. In brief, after separated with SDS-PAGE gel, total cellular proteins were transferred to a polyvinylidene fluoride (PVDF) membrane (Millipore, ISEQ00010, USA). The PVDF membrane was then incubated overnight at 4 °C with various antibodies (Supplementary Table 3). The ECL Western Blotting Substrate (Pierce, 32,106) was used to visualize target proteins.

### Turnover assays

The stably *LINC00240-*OE, *LINC00240*-KD or control HGC-27 or MGC80-3 cells were seeded into 6-well plates and then cultured for 24 h. Cycloheximide (CHX) (Merck, # 66–81-9, US) at a final concentration of 200 μg/mL was added to the cells to stop de novo protein synthesis. The HGC-27 or MGC80-3 cells were harvested at the indicated times after CHX treatments. Western blot was performed to measure the DDX21 and GAPDH protein levels in HGC-27 or MGC80-3 cells.

### Ubiquitination assays

As reported previously, gastric cancer cells were firstly transfected with the pcDNA3.1-HA-ubiquitin (HA-ubi) plasmid [15, 23]. At 36 h after transfection, MG132 at a final concentration of 50 μg/mL was added to the cells and incubated for 6 h. To isolate ubiquitinated DDX21, proteins in the cell lysate were immunoprecipitated with anti-DDX21 antibody and then detected with the anti-HA antibody by Western blot.

### Statistics

Student’s *t* test was used for calculation of the difference between two groups. Impacts of *LINC00240* expression on gastric cancer patients’ survival were tested by Kaplan–Meier plots and survival durations were analyzed using the log-rank test. A *P* value of less than 0.05 was used as the criterion of statistical significance. All analyses were performed with SPSS software package (Version 16.0, SPSS Inc.) or GraphPad Prism (Version 8.0, GraphPad Software, Inc.).

## Results

### Increased expression of LINC00240 at chromosome 6p22.1 locus in gastric cancer tissues was associated with shortened survival time of patients

**Fig. 1:**
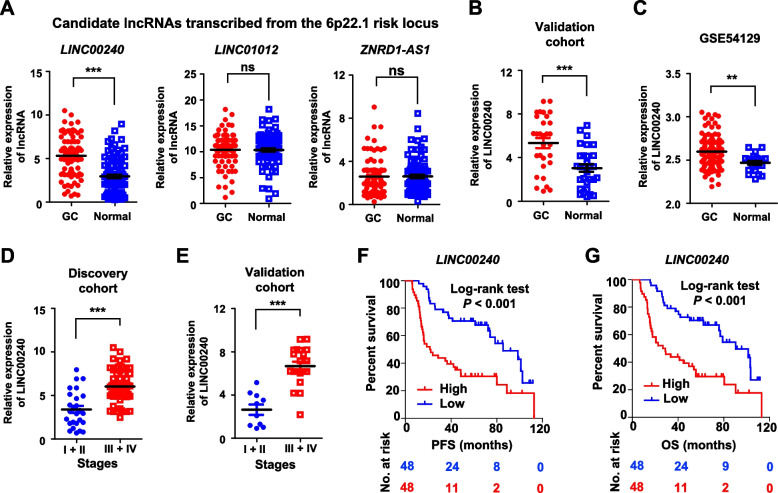
*LINC00240* is a significantly elevated lncRNA in gastric cancer specimens and associated with shortened survival time of patients. **A** Relative expression of three candidate lncRNAs transcribed from the 6p22.1 risk locus (*LINC00240*, *LINC01012*, and *ZNRD1-AS1*) in paired gastric cancer specimens and normal tissues of Discovery cohort (*n* = 96). **B**, **C** Significantly upregulated expression of *LINC00240* in gastric cancer specimens of Validation cohort (*n* = 30) and another Chinese gastric cancer cohort (GSE54129) compared to normal tissues. **D**, **E** In Discovery cohort and Validation cohort, there were much higher *LINC00240* expression levels in cancerous tissues from gastric cancer patients with high-grade diseases compared to malignant tissues from patients with low-grade diseases. **F**, **G** The gastric cancer patients with higher *LINC00240* expression had a shorter PFS or OS compared to patients with lower *LINC00240* expression (Discovery cohort). Data are shown as mean ± SD. **P < 0.01; ***P < 0.001 by paired Student’s *t* test (**A**, **B**), or by unpaired Student’s *t* test (**C**, **D**, **E**). ns: not significant. Log-rank test was used for survival comparison (**F**, **G**)

To examine whether lncRNAs transcribed from the chromosome 6p22.1 risk locus contribute to gastric cancer development, we firstly measured levels of three candidate lncRNAs (*LINC00240*, *LINC01012*, and *ZNRD1-AS1*) in paired gastric cancer specimens and normal tissues of Discovery cohort (*n* = 96) (Fig. 1A). We found that only *LINC00240* showed evidently elevated expression in malignant tissues compared to normal stomach specimens (*P* < 0.001) (Fig. 1A). In line with this, we found markedly increased levels of *LINC00240* in gastric cancer specimens of Validation cohort (*n* = 30) (both *P* < 0.001) (Fig. 1B). In support of this notion, significantly increased *LINC00240* expression in cancerous specimens was observed compared to normal tissues in another gastric cancer cohort from China (GSE54129) (*P* < 0.01). There was remarkably elevated expression of *LINC00240* in patients with advanced stage diseases compared to cases with early-stage diseases (Fig. 1D and E). Importantly, *LINC00240* levels were significantly associated with progression-free survival (PFS) (log-rank *P* < 0.001; Cox regression *P* = 3.9 × 10^–4^) and overall survival (OS) in gastric cancer patients of Discovery cohort (log-rank *P* < 0.001; Cox regression *P* = 2.6 × 10^–4^) (Fig. 1F and G). Patients with lower *LINC00240* expression had prolonged time of PFS or OS compared to ones with high *LINC00240* levels (Fig. 1F and G), suggesting that *LINC00240* might be involve in gastric cancer progression as a novel oncogene.

### LINC00240 enhanced malignant proliferation of gastric cancer cells in vitro and in vivo

**Fig. 2:**
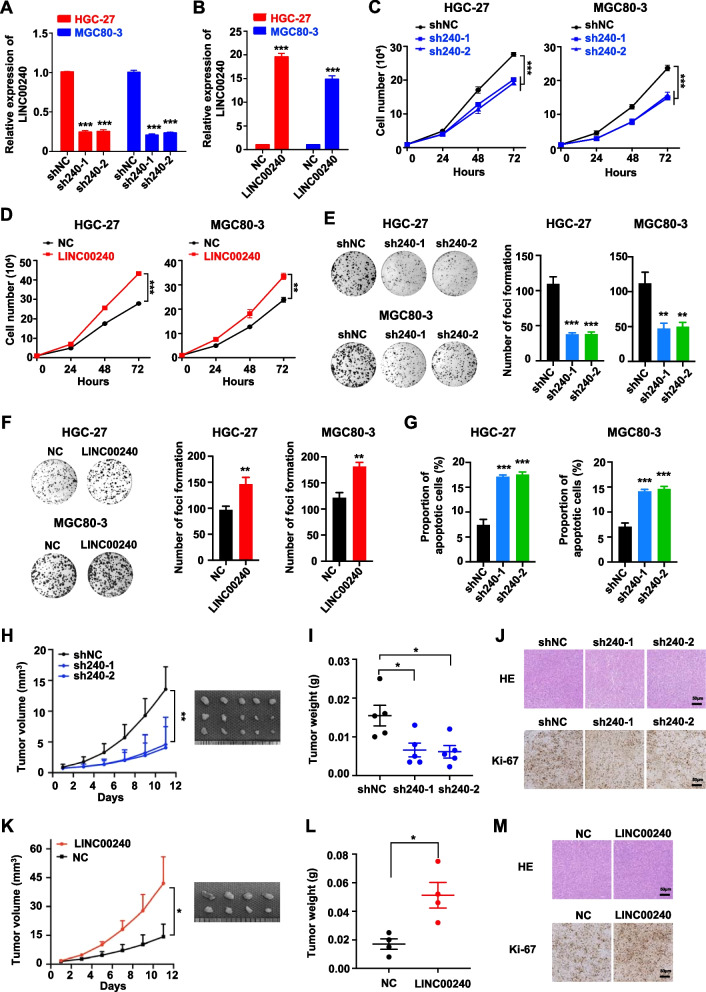
*LINC00240* promotes malignant proliferation of gastric cancer cells in vitro and in vivo. **A**, **B** Relative expression of *LINC00240* in HGC-27 and MGC80-3 gastric cancer cell lines with stably silenced *LINC00240* (shL240-1 or shL240-2) or with stably forced-expressed *LINC00240* (LINC00240). **C** Silencing of *LINC00240* significantly repressed viability of HGC-27 and MGC80-3 cells*.* **D** Overexpression of *LINC00240* significantly promoted proliferation of gastric cancer cells*.* **E**, **F** In colony formation assays, silencing of *LINC00240* markedly inhibited clone formation of HGC-27 and MGC80-3 cells. Overexpressed *LINC00240* remarkably increased clonogenicity of gastric cancer cells. **G** Silencing of *LINC00240* significantly promoted apoptosis of gastric cancer cells. **H**-**J** Evidently decreased growth and tumor weights of the HGC-27 xenografts with stably depleted *LINC00240* were observed compared to the controls. **K**-**M** The xenografts stably over-expressing *LINC00240* grew much faster and showed an obvious increase in tumor weight compared to the control xenografts. Data are shown as mean ± SD. *P < 0.05; **P < 0.01; ***P < 0.001 by unpaired Student’s *t* test (**A**-**J**)

We next examined the involvement of *LINC00240* in gastric cancer development in vitro and in vivo. We firstly examined levels of *LINC00240* in human GES-1, MKN-28, MKN-45, AGS, BGC-823, HGC-27 and MGC-803 cell lines. Evidently higher *LINC00240* expression levels in gastric cancer cells (MKN-28, MKN-45, AGS, BGC-823, HGC-27 and MGC-803) were observed than that in normal human GES-1 cells (all *P* < 0.001) (Supplementary Fig. 1). However, there were no obvious *LINC00240* expression differences among various gastric cancer cell lines. Therefore, we chose HGC-27 and MGC-803 cell lines to explore the role of *LINC00240*. Multiple gastric cancer cell lines were then generated via stably silencing of *LINC00240* by shRNAs or forced-expressing the lncRNA by the *LINC00240* plasmid. These gastric cancer cells successfully transduced by lentivirus were selected by blasticidin or puromycin. In HGC-27 and MGC80-3 cells stably expressing *LINC00240* shRNAs, there was significantly decreased expression of the lncRNA (shL240-1 or shL240-2 vs. shNC: all *P* < 0.001) (Fig. 2A). Strikingly over-expressed *LINC00240* was found in HGC-27 and MGC80-3 cells stalely expressing the *LINC00240* construct (LINC00240 vs. NC: both *P* < 0.001) (Fig. 2B). As shown in Fig. 2C and D, silencing of *LINC00240* significantly inhibited proliferation of HGC-27 and MGC80-3 cells (all *P* < 0.001); whereas, ectopic *LINC00240* expression markedly promoted viability of gastric cancer cells (both *P* < 0.01). Consistently, knocking-down expression of *LINC00240* suppressed clone formation of HGC-27 and MGC80-3 cells (all *P* < 0.01) (Fig. 2E). Gastric cancer cells with overexpressed *LINC00240* showed reinforced clonogenicity (both *P* < 0.01) (Fig. 2F). Moreover, silencing of *LINC00240* significantly promoted apoptosis of gastric cancer cells, but did not impact cell cycle of gastric cancer cells (Fig. 2G and Supplementary Fig. 2). Together, these data elucidated the oncogenic functions of *LINC00240* in gastric cancer.

We then assessed the in vivo role of LINC00240 using gastric cancer xenografts. Importantly, stable silencing of *LINC00240* evidently inhibited growth of the gastric cancer xenografts compared to the controls (*P* < 0.05) (Fig. 2H-J). On the contrary, xenografts stably over-expressing *LINC00240* grew much faster and showed an evident increase in tumor volume and tumor weight compared to the control xenografts (*P* < 0.05) (Fig. 2K-M). In addition, we also detected expression levels of multiple apoptotic proteins (BAX, BAK and BCL-2) in gastric cancer xenografts, which indicated the pro-apoptotic of *LINC00240* (Supplementary Fig. 3A). Collectively, these results suggested that LINC00240 could promote malignant proliferation of gastric cancer in vivo.

### LINC00240 improved invasive activities of gastric cancer cells

**Fig. 3:**
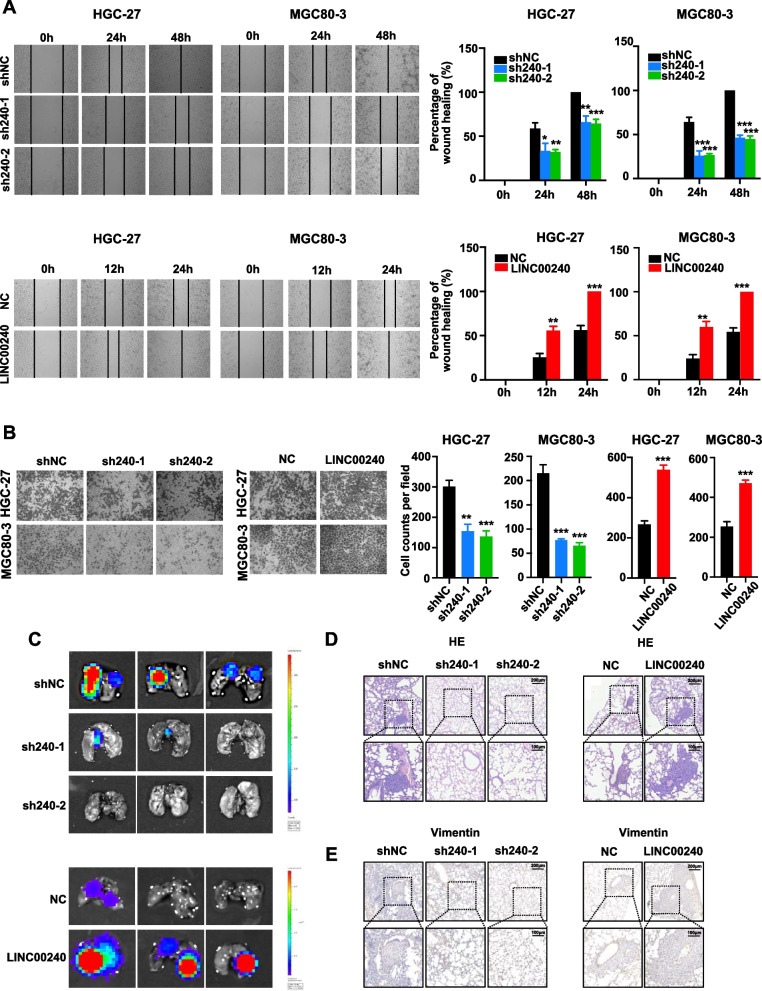
*LINC00240* enhances migration and invasion capabilities of gastric cancer cells. **A** In HGC-27 and MGC80-3 gastric cancer cells, *LINC00240*-knockdown inhibited wound-healing and the stably enforced *LINC00240* expression accelerated wound-healing. The lines are the edges of cell layers. **B** *LINC00240*-knockdown reduced invasion of gastric cancer cells. Overexpression of *LINC00240* promoted invasion abilities of HGC-27 and MGC80-3 cells. **C** Representative fluorescent images of metastatic tumors in lungs of nude mice. **D** Representative images of HE slides of lung metastases. **E** Representative images of IHC staining showing the expression of Vimentin within lung metastases. Data are shown as mean ± SD. *P < 0.05; **P < 0.01; ***P < 0.001 by unpaired Student’s *t* test (**A**-**F**)

We next investigated if *LINC00240* influences the migration and invasion of gastric cancer cells (Fig. 3). The wound-healing assays were performed to determine the impact of *LINC00240* on mobility of gastric cancer cells. Stable *LINC00240*-KD significantly impaired HGC-27 cell motility, whereas stabilized *LINC00240*-OE accelerated migration capability of gastric cancer HGC-27 cells. In line with this observation, silencing of *LINC00240* evidently weakened gastric cancer MGC80-3 cell migration and forced-expressed *LINC00240* promoted motility capability of MGC80-3 cells (Fig. 3A). The Matrigel invasion assays elucidated that stably silenced *LINC00240* could reduce invasion of gastric cancer cells. In contrast, increased invasion capability of gastric cancer cells was observed after ectopic *LINC00240* expression (Fig. 3B). We then examined if *LINC00240* could regulate metastasis of gastric cancer cells in vivo. To establish metastasis mice models, stably *LINC00240*-KD HGC-27 cells, stably *LINC00240*-OE cells or NC cells were injected into the tail vein of mice. We found that *LINC00240*-depletion inhibited lung metastasis of gastric cancer cells; whereas, stabilized *LINC00240* overexpression obviously enhanced gastric cancer lung metastases (Fig. 3C). HE staining and vimentin IHC further confirmed the effect of *LINC00240* expression on metastasis of gastric cancer cells in vivo (Fig. 3D and E). These results indicated that *LINC00240* enhances migration and invasion capability of gastric cancer cells.

### LINC00240 interacted with oncoprotein DDX21 in gastric cancer cells

**Fig. 4:**
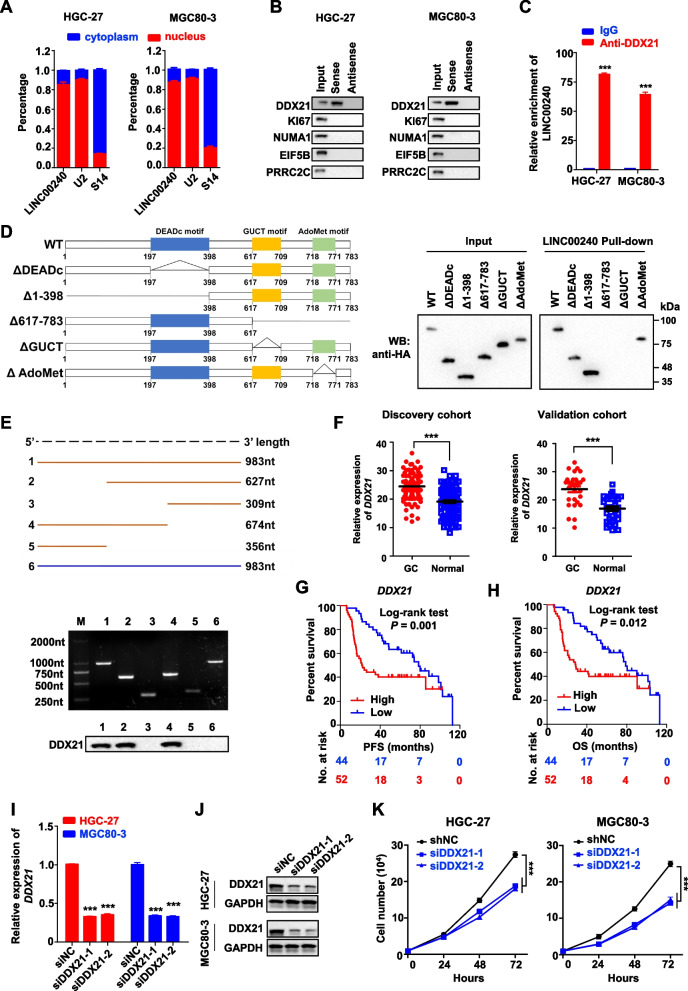
LINC00240 interacts with oncoprotein DDX21 in gastric cancer cells. **A** LINC00240 predominantly locates in the nuclear fraction of gastric cancer cells. **B** LINC00240 pull-down followed by Western blot validated its interaction with DDX21 and other candidate proteins identified by Mass spectrometry. **C** LINC00240 could be precipitated with antibody against DDX21 as compared with the IgG control in HGC-27 and MGC80-3 cells. Relative enrichment (means ± SD) represents RNA levels associated with DDX21 relative to an input control from three independent experiments. **D** Diagrams of HA-tagged DDX21 and its truncated forms used in LINC00240 pull-down assays. Western blot of HA-tagged wild-type (WT) DDX21 and its truncated forms retrieved by in vitro transcribed biotinylated LINC00240. **E** Truncated LINC00240 mapping of DDX21 binding domain. Top panel: schematic diagrams of LINC00240 full-length and truncated fragments. Middle panel: RNA sizes of in vitro transcribed full-length and truncations of LINC00240. Bottom panel: Western blot of DDX21 pulled down by various LINC00240 fragments. **F** There were evidently increased *DDX21* expression in gastric cancer tissues compared to normal tissues in both Discovery cohort and Validation cohort*.* **G**, **H** The high expression levels of *DDX21* were significantly associated with poor OS or PFS of gastric cancer patients. **I**, **J** Silencing of *DDX21* expression with different siRNAs in HGC-27 and MGC80-3 cells. **K** *DDX21*-knockdown significantly suppressed proliferation of HGC-27 and MGC80-3 gastric cancer cell lines. Data are shown as mean ± SD. ***P < 0.001 by unpaired Student’s *t* test (**C**, **F**, **J**, **K**)

To explore how *LINC00240* contributing to gastric tumorigenesis, we firstly detected the cellular localization of LINC00240 in gastric cancer cells. Interestingly, we found that LINC00240 locates dominantly in nucleus of gastric cancer cells (Fig. 4A). Accumulating evidences demonstrated that lncRNAs could function as protein-binding scaffolds in various malignancies [15, 23, 26, 27]. To test if LINC00240 interacts with certain proteins in gastric cancer, we then performed RNA pulldown assays plus mass spectrometry proteomics with the MGC80-3 cell extracts pulled-down by LINC00240. Among proteins pulled-down by LINC00240 (Supplementary Table 4), we successfully verified DDX21 as the lncRNA-binding protein through independent RNA pulldown assays and Western blot in HGC-27 or MGC80-3 cells (Fig. 4B). Nevertheless, other four cancer-related candidate proteins (KI67, NUMA1, EIF5B and PRRC2C) could not be validated in cells (Fig. 4B). Importantly, RIP assays indicated that there was noticeable enrichment of LINC00240 in RNA–protein complexes precipitated with the anti-DDX21 antibody in both gastric cancer cell lines as compared with the IgG control (*P* < 0.001) (Fig. 4C). To explore the specific regions or domains required for the interaction between LINC00240 and DDX21, we then constructed various truncated *DDX21* constructs and truncated *LINC00240* plasmids (Fig. 4D and E). The results elucidated that the GUCT motif (aa 617–709) of DDX21 protein and the middle region of LINC00240 RNA (nucleotides 357–674) are required for the interaction between LINC00240 and the protein (Fig. 4D and E).

DDX21 is a crucial DEAD cassette RNA helicase and acts as an oncogene in multiple cancers [28–34]. Indeed, significantly elevated *DDX21* expression in gastric cancer tissues was also observed compared to normal tissues in both Discovery cohort and Validation cohort (*P* < 0.001) (Fig. 4F). Aberrantly high expression of *DDX21* was associated with poor OS or PFS of gastric cancer patients (log-rank *P* = 0.001 or 0.012; Cox regression *P* = 0.029 or 0.027) (Fig. 4 G and H). In line with these data, silencing of *DDX21* profoundly inhibited viability of HGC-27 or MGC80-3 cells (Fig. 4I-K), indicating the oncogenic nature of *DDX21* in gastric cancer. Collectively, these findings suggested that LINC00240 could interact with oncoprotein DDX21 via the GUCT motif in gastric cancer cells.

### LINC00240 repressed DDX21 degradation via the ubiquitin–proteasome pathway

**Fig. 5:**
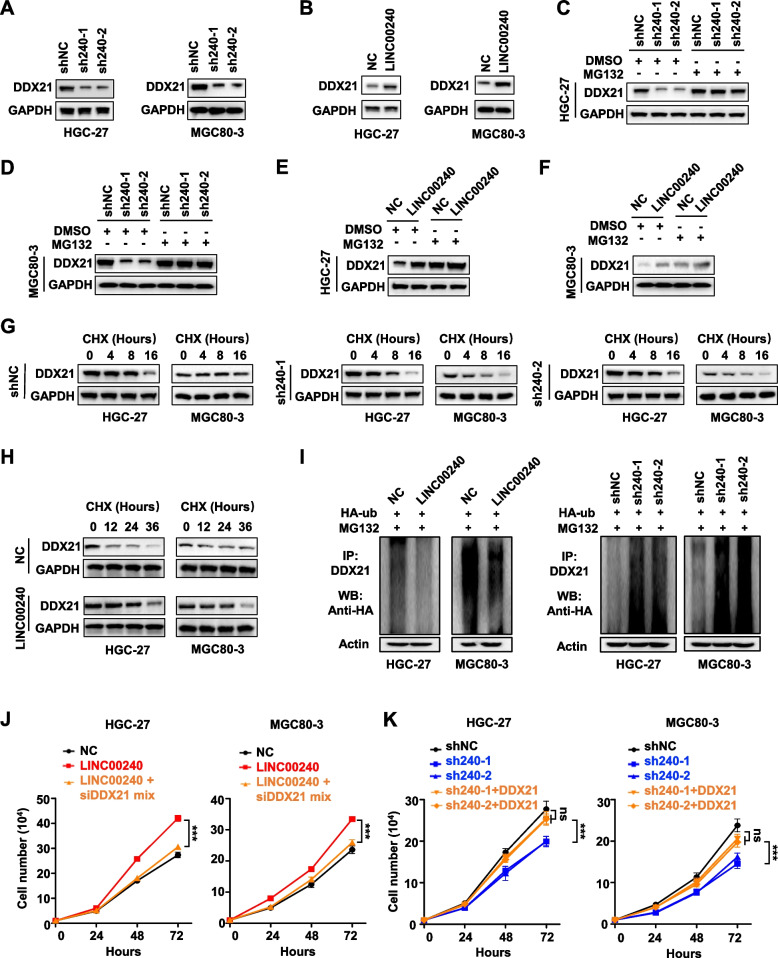
LINC00240 represses DDX21 degradation via the ubiquitin–proteasome pathway. **A**, **B** Western blot analyses of DDX21 protein in cells with stable knock-down of *LINC00240* or enforced expression of *LINC00240.* **C**, **D** Treatment of the stably *LINC00240*-knock-down gastric cancer cells with the 26S protostome inhibitor MG132 elevated expression of DDX21 protein. **E**, **F** Treatment of gastric cancer cells with MG132 eliminated LINC00240-induced upregulation of DDX21 protein expression. **G**, **H** Gastric cancer cells with stable *LINC00240*-knockdown, overexpression of *LINC00240* and control cells were treated with cycloheximide (CHX) for the indicated periods of time. **I** Western blot of the ubiquitination of DDX21 in cells that stabilized silenced *LINC00240* or over-expressed *LINC00240*. **J** Silencing of *DDX21* with siRNAs significantly inhibited proliferation of HGC-27 or MGC80-3 cells with stably overexpressed *LINC00240*. **J** Over-expression of *DDX21* could enhance growth of gastric cancer cells with stable silencing of *LINC00240* with shRNAs. Data are shown as mean ± SD. ***P < 0.001 by unpaired Student’s *t* test (**J**, **K**). ns: not significant

Intriguingly, silencing of *LINC00240* evidently suppressed DDX21 protein expression in gastric cancer cells (Fig. 5A). On the contrary, over-expressed *LINC00240* markedly up-regulated DDX21 protein levels in HGC-27 or MGC80-3 cells (Fig. 5B). Treatment of the stably *LINC00240*-KD gastric cancer cells with the 26S protostome inhibitor MG132 increased expression of endogenous DDX21 protein compared to the controls (Fig. 5 C and D). In contrast, MG132 abolished LINC00240-induced up-regulation of DDX21 protein in HGC-27 or MGC80-3 cells (Fig. 5 E and F), indicating that the lncRNA might participate in regulating the proteasome degradation of DDX21. To verify this, we next examined DDX21 expression in HGC-27 or MGC80-3 cells treated with the protein synthesis inhibitor CHX. It was found that the DDX21 protein levels declined much faster in the stably *LINC00240*-KD cells than those in the control cells (Fig. 5G). Conversely, treatment of the stably *LINC00240-*OE gastric cancer cells with CHX led to an evidently longer half-life of DDX21 protein than in control cells (Fig. 5H). We then explored whether *LINC00240*-dependent degradation of DDX21 was mediated by its ubiquitination. After endogenous DDX21 was immunoprecipitated in cells transfected with HA-Ub, strikingly increased ubiquitin signals of DDX21 protein were detected in the stably *LINC00240*-KD cells in comparison to the control cells (Fig. 5I). In line with this, the ubiquitination of DDX21 was evidently decreased in the stably *LINC00240-*OE cells compared to the control cells (Fig. 5I). Rescue assays indicated that silencing of *DDX21* with siRNAs significantly inhabited proliferation of gastric cancer cells with stably overexpressed *LINC00240* (both *P* < 0.001) (Fig. 5J). On the contrary, over-expression of *DDX21* could enhance cell proliferation of gastric cancer cells with stable silencing of *LINC00240* with shRNAs (all *P* > 0.05) (Fig. 5K). Taken together, these results elucidated that LINC00240 stabilize DDX21 protein via the ubiquitin–proteasome system.

### LINC00240 interrupts binding of DDX21 with its novel deubiquitinase USP10

**Fig. 6:**
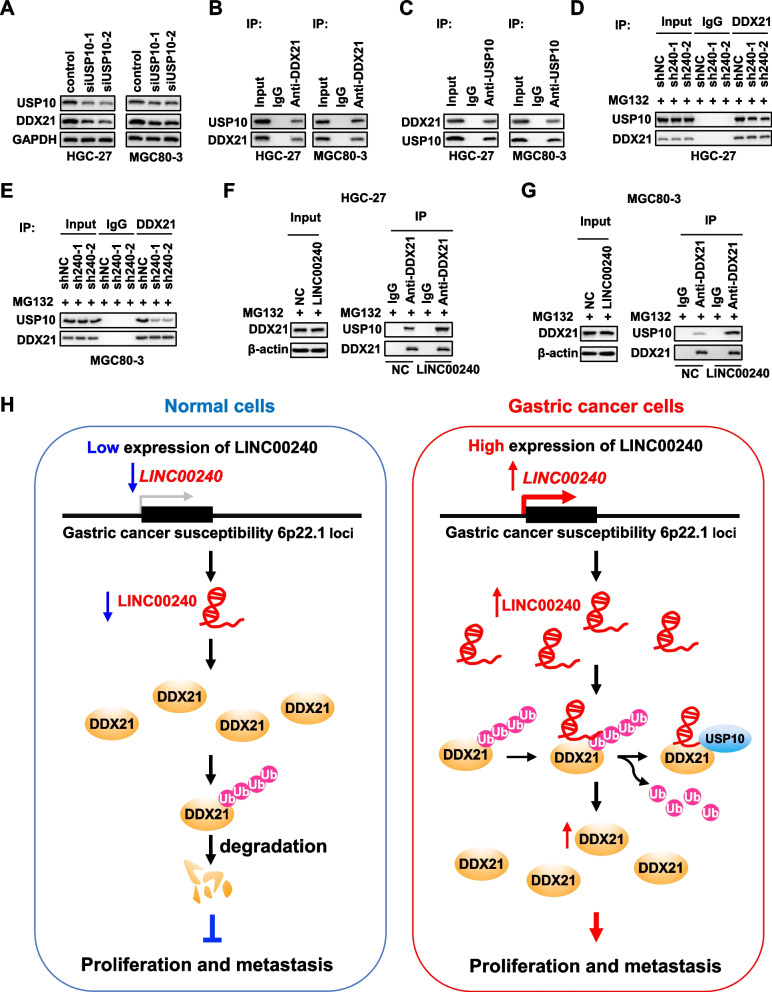
LINC00240 facilitates the interaction between DDX21 and the deubiquitinase USP10. **A** Silencing of *USP10* significantly suppressed DDX21 protein expression in gastric cancer cells. **B**, **C** Interactions between USP10 and DDX21 in gastric cancer cells were verified via Co-IP assays. **D**, **E** Silencing of *LINC00240* diminished the interaction between USP10 and DDX21 in HGC-27 and MGC80-3 cells. **F**, **G** Overexpressed *LINC00240* strengthened the interaction between USP10 and DDX21. **H** Diagram depicted how LINC00240 promotes DDX21 stabilization via intensifying interactions between DDX21 and its novel deubiquitinase USP10

To reveal how LINC00240 retards the ubiquitin–proteasome degradation of DDX21, we analyzed proteins pulled-down by LINC00240 in MGC80-3 cells. Mass spectrometry indicated that there was only one deubiquitinase, USP10. To confirm if USP10 is a novel deubiquitinase of DDX21, we firstly examined DDX21 levels in HGC-27 or MGC80-3 cells with silenced expression of *USP10* (Fig. 6A). After knock-downing of USP10 expression, obviously decreased DDX21 protein levels could be detected in gastric cancer cells in comparation with the control cells (Fig. 6A). Importantly, Co-IP assays showed that endogenous USP10 could be precipitated with DDX21 in HGC-27 or MGC80-3 cells (Fig. 6B). Endogenous DDX21 could also be precipitated with USP10 in both gastric cancer cell lines (Fig. 6C). These data elucidated that USP10 might be a potential deubiquitinase of DDX21 in gastric cancer. We next investigated if LINC00240 impacts interactions between DDX21 and USP10 in cells. Less USP10 protein could be precipitated with DDX21 in the stably *LINC00240*-KD cells compared to the control cells (Fig. 6 D and E). In contrast, more USP10 protein could be precipitated with DDX21 in the stably *LINC00240-*OE cells compared to the control gastric cancer cells (Fig. 6 D and G). Furthermore, silencing of *LINC00240* evidently suppressed DDX21 protein expression in gastric cancer xenografts (Supplementary Fig. 3B). However, *LINC00240* did not impact the expression level of USP10 protein in xenografts (Supplementary Fig. 3B). Together, these findings suggested that LINC00240 promotes DDX21 stabilization via intensifying interactions between DDX21 and its novel deubiquitinase USP10 in gastric cancer (Fig. 6H).

## Discussion

In this study, we identified a novel gastric cancer susceptibility gene, *LINC00240*, in the 6p22.1 risk locus. There were significantly higher *LINC00240* expression levels in gastric cancer tissues as compared with normal specimens. High levels of *LINC00240* are associated with evidently shortened OS or PFS time of gastric cancer patients. *LINC00240* functions as an oncogene to enhance malignant behaviors of gastric cancer cells in vitro and in vivo. We also found that LINC00240 interacts with oncoprotein DDX21, suppresses its ubiquitination and stabilize the protein via intensifying binding of DDX21 with its novel deubiquitinase USP10, which, thus, leading to disease progression of gastric cancer (Fig. 6H). This line of research provides compelling evidences which contributing to the understanding of the genetic component and pathogeneses of gastric cancer.

GWAS have identified many independent genetic signals associated with cancer risk [35]. However, understanding the mechanisms by which these susceptibility genomic loci have an impact on associated cancers remains a great hurdle. Multiple lines of evidences indicated that lncRNAs are transcribed from these cancer-risk loci and confer to tumorigenesis [17–20, 36–39]. For instance, *PCAT1*, *PVT1*, and *PCAT19* in prostate cancer, *CUPID1* and *CUPID2* in breast cancer, *PTCSC2* and *PTCSC3* in thyroid cancer, *LINC00673* in pancreatic cancer, *lncPSCA* in gastric cancer, as well as *LCETRL3* and *LCETRL4* in non-small cell lung cancer, are implicated in malignant development. Guo et al. found that lncRNA *PCAT1* binds AR and LSD1 and is essential for their recruitment to the enhancers of *GNMT* and *DHCR24*, two important genes in prostate cancer development and progression [16]. Another such example is lncRNA *PTCSC2* which interacts with MYH9 and, thus, reverses MYH9-mediaed inhibition of activities of a bidirectional promoter shared by *FOXE1* and *PTCSC2* in thyroid cancer [38]. Our study, for the first time, indicated that *LINC00240* is a novel gastric cancer susceptibility gene transcribed from the GWAS-identified 6p22.1 locus, highlighting the functional impotence of lncRNAs at risk loci during cancer progression.

DEAD-box RNA helicases are crucial for regulating various RNA metabolism. DDX21 is a member of the DEAD-box RNA helicase family that can promote malignant behaviors via various mechanisms [28–34]. DDX21 controls ribosome biogenesis via sensing the transcriptional status of both RNA Pol I and II in cells [28, 34]. That is, DDX21 interacts with 7SK RNA and is recruited to the promoters of genes encoding ribosomal proteins as well as snoRNAs to enhance their transcription [28, 34]. Consistently, DDX21 could stimulate breast cancer cell proliferation through activation of PARP-1, rDNA transcription, ribosome biogenesis, and protein translation [30]. For a number of biological processes, maintenance of sufficient nucleotide pools is vital. Alterations during nucleotide metabolism may lead to malignant transformation and tumorigenesis [31]. Intriguingly, knockdown of *ddx21* in zebrafishes confers resistance to nucleotide depletion. DDX21 acts as a sensor of nucleotide stress and contributes to melanoma development [31]. Furthermore, DDX21 plays its oncogenic role in colorectal cancer via inducing genome fragility, damaging double-strand repair and procrastinating HR repair [33]. In line with these findings, we also found that the high expression of DDX21 in gastric cancer leads to poor prognosis and disease progression of gastric cancer patients.

Emerging evidence demonstrates that deubiquitylases significantly contribute to a number of malignancies including gastric cancer. As a deubiquitinase essential for the deubiquitylation, USP10 controls turnover and function of many substrates including p53, H2A.Z, SIRT6, NEMO, TOP2α, AMPK, p14ARF, FLT3, KLF4, YAP/TAZ, NICD1 and MSH2 [40–51], which play key roles in multiple oncogenic signaling pathways. For example, USP10 is the important deubiquitinase required to stabilize oncogenic forms of the kinase FLT3, a critical therapeutic target in acute myeloid leukemia (AML) [47]. Targeting of USP10 with its small-molecule inhibitors showed efficacy in preclinical models of mutant-FLT3 AML [47]. Similarly, USP10 acts as a YAP/TAZ-activating deubiquitinase through directly binding and reverting their proteolytic ubiquitination [49]. USP10 defects improved polyubiquitination of YAP/TAZ and their proteasomal degradation in hepatocellular carcinoma [49]. Interestingly, in the current study, we also identified USP10 as a binding partner for both DDX21 and LINC00240. Additionally, we for the first time reported that LINC00240 enhances USP10-mediated deubiquitylation of DDX21 and evaluates DDX21 levels.

In summary, we revealed the importance of *LINC00240* transcribed from the 6p22.1 locus in gastric cancer and uncovered a novel regulatory partnership between *LINC00240* and USP10 in controlling ubiquitination of oncogenic protein DDX21. Given their vital roles and high abundances in gastric cancer, the *LINC00240*-USP10-DDX21 axis could be a potential therapeutic target for the lethal disease in the clinic.

## Supplementary Information

**Additional file 1:** **Supplementary Table 1.** Primers for RT-qPCR. **Supplementary Table 2.** Sequences of shRNAs and siRNAs. **Supplementary Table 3.** Antibodies used in the study. **Supplementary Table 4.** Mass spectrometry of proteins pulled-down by LINC00240 in MGC80-3 cells

**Additional file 2:** **Supplementary Figure 1.** The relative expression levels of *LINC00240* in human GES-1, MKN-28, MKN-45, AGS, BGC-823, HGC-27 and MGC-803 cell lines. ***P < 0.001. **Supplementary Figure 2.** Silencing of *LINC00240* significantly promoted apoptosis of gastric cancer cells (A), but did not impact cell cycle (B). **Supplementary Figure 3. **Expression of apoptotic proteins (A), DDX21 and USP10 (B) in gastric cancer xenografts.

## Data Availability

All data are included in the manuscript. Materials used in the current study are available from the corresponding author on reasonable requests.

## Abbreviations

*H. pylori*: *Helicobacter pylori*
GWASs: Genome-wide association studies
lncRNAs: Long noncoding RNAs
Pol II: RNA polymerase II
FBS: Fetal bovine serum
RT-qPCR: Quantitative reverse transcription PCR
OE: Overexpression
KD: Knockdown
LS-MS/MS: Liquid chromatography-tandem mass spectrometry
RNA-IP: RNA immunoprecipitation
PVDF: Polyvinylidene fluoride
CHX: Cycloheximide
HA-ubi: HA-ubiquitin
PFS: Progression-free survival
OS: Overall survival
AML: Acute myeloid leukemia
