# Supplementary material for: LINC00240 in the 6p22.1 risk locus promotes gastric cancer progression through USP10-mediated DDX21 stabilization
Source: J Exp Clin Cancer Res. 2023 Apr 18;42:89. doi: 10.1186/s13046-023-02654-9 (PMC10111703; doi:10.1186/s13046-023-02654-9)
Supplement: Supplementary file 1 — Additional file 1: Supplementary Table 1. Primers for RT-qPCR. Supplementary Table 2. Sequences of shRNAs and siRNAs. Supplementary Table 3. Antibodies used in the study. Supplementary Table 4. Mass spectrometry of proteins pulled-down by LINC00240 in MGC80-3 cells [file 13046_2023_2654_MOESM1_ESM.docx]

**Supplementary Table 1. Primers for RT-qPCR**

| Name | Primer Sequence |
| --- | --- |
| LINC00240-F | AACAAAGTCCCTTGATTTAGAC |
| LINC00240-R | CAGAGGCAGCCAGACAAC |
| LINC01012-F | CTGACGCAGGTGGACAAG |
| LINC01012-R | AACCCTAAATGACGCTGA |
| ZNRD1ASP-F | ATGCCCAGGATGAGGTGA |
| ZNRD1ASP-R | GCTGTGCAGGATACGAGGT |
| DDX21-F | TCATCAAGGACGCACTATCATCT |
| DDX21-R | CCTTTCAGGGTGATTTCCCTTT |
| S14-F | GGCAGACCGAGATGAATCCTC |
| S14-R | CAGGTCCAGGGGTCTTGGTCC |
| U2-F | CATCGCTTCTCGGCCTTTTG |
| U2-R | TGGAGGTACTGCAATACCAGG |
| β-actin-F | GGCGGCACCACCATGTACCCT |
| β-actin -R | AGGGGCCGGACTCGTCATACT |

**Supplementary Table 2. Sequences of shRNAs and siRNAs**

| Name | shRNA or siRNA Sequence |
| --- | --- |
| sh240-1 | ACCGGTCGGCCTAGAGACTGCGATGGTTCTCGAGAACCATCGCAGTCTCTAGGTTTTTGAATTC |
| sh240-2 | ACCGGTCGGGCCTCTGCTGTCTACAGATCTCGAGATCTGTAGACAGCAGAGGCTTTTTGAATTC |
| shNC | CCGGTAGTCGCATACGGAACATTCGCTCGAGCGAATGTTCCGTATGCGACTATTTTTG |
| siDDX21-1 | Sense 5'-CCGCAUCAGUAACAGAAAUTT-3' |
|  | Antisense 5'-AUUUCUGUUACUGAUGCGGTT-3' |
| siDDX21-2 | Sense 5'-GCUCCUUGAUCAACUCAAATT-3' |
|  | Antisense 5'-UUUGAGUUGAUCAAGGAGCTT-3' |
| siUSP10-1 | Sense 5'-CCUGUGGACUUGGAAAUUATT-3' |
|  | Antisense 5'-UAAUUUCCAAGUCCACAGGTT-3' |
| siUSP10-2 | Sense 5'-GCAGGUUGAAGUCAAAGAATT-3' |
|  | Antisense 5'-UUCUUUGACUUCAACCUGCTT-3' |
| NC | Sense 5'-UUCUCCGAACGUGUCACGUTT-3' |
|  | Antisense 5'-ACGUGACACGUUCGGAGAATT-3' |

**Supplementary Table 3. Antibodies used in the study**

| Name | Company | Catalog Number |
| --- | --- | --- |
| DDX21 | Proteintech | 10528-1-AP |
| KI67 | Abcam | ab92742 |
| NUMA1 | Abcam | ab10926 |
| EIF5B | Proteintech | 13527-1-AP |
| PRRC2C | Abcam | ab117790 |
| USP10 | Abcam | ab109219 |
| IgG Isotype Control | Invitrogen | 02-6102 |
| HA-tag | Abcam | ab236632 |
| β-actin | Abcam | ab8227 |
| GAPDH | Proteintech | 10494-1-AP |
| Anti-rabbit IgG, HRP-linked antibody | CST | 7074s |
| Anti-mouse IgG, HRP-linked antibody | CST | 7076s |

**Supplementary Table 4.** Mass spectrometry of proteins pulled-down by LINC00240 in MGC80-3 cells

| No. | Gene names | Unique peptides | LFQ intensity Sense | LFQ intensity Antisense |
| --- | --- | --- | --- | --- |
| 1 | KI67 | 34 | 205600000 | 0 |
| 2 | DDX21 | 17 | 301080000 | 0 |
| 3 | NUMA1 | 16 | 87510000 | 0 |
| 4 | EIF5B | 16 | 199280000 | 0 |
| 5 | PRRC2C | 16 | 154610000 | 0 |
| 6 | TOP2A | 16 | 348660000 | 0 |
| 7 | MDC1 | 14 | 131200000 | 0 |
| 8 | LMNA | 13 | 103970000 | 0 |
| 9 | NUFIP2 | 13 | 192830000 | 0 |
| 10 | KTN1 | 12 | 55703000 | 0 |
| 11 | CKAP5 | 12 | 104910000 | 0 |
| 12 | PA2G4 | 11 | 334550000 | 0 |
| 13 | PRRC2A | 11 | 86227000 | 0 |
| 14 | LARS1 | 10 | 60254000 | 0 |
| 15 | RBMX | 10 | 190650000 | 0 |
