# Supplementary figures and images for: LINC00240 in the 6p22.1 risk locus promotes gastric cancer progression through USP10-mediated DDX21 stabilization
Source: J Exp Clin Cancer Res. 2023 Apr 18;42:89. doi: 10.1186/s13046-023-02654-9 (PMC10111703; doi:10.1186/s13046-023-02654-9)

# Supplementary Figure 1

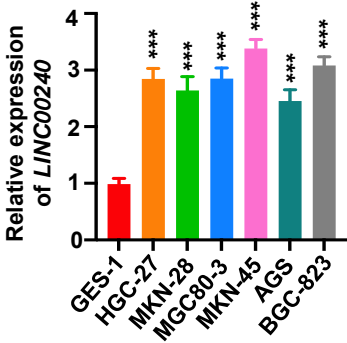

Supplementary Figure 2

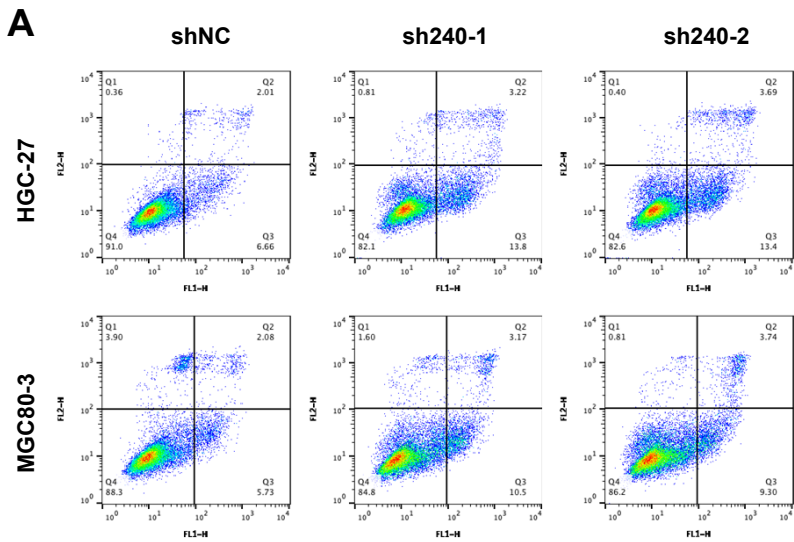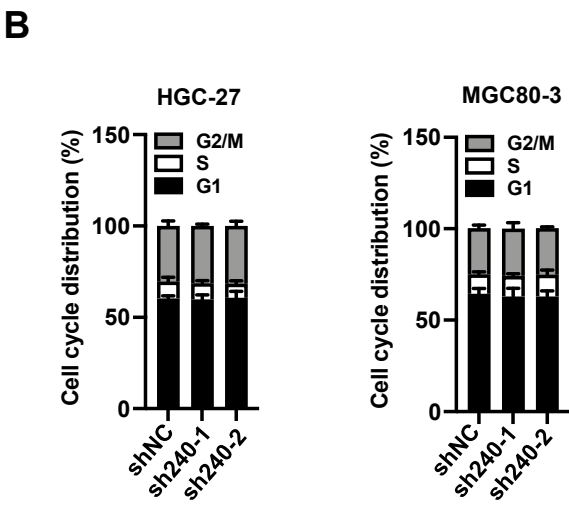

# Supplementary Figure 3

A

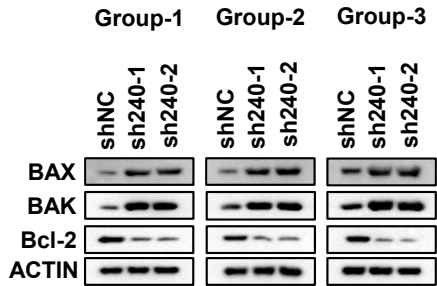

B

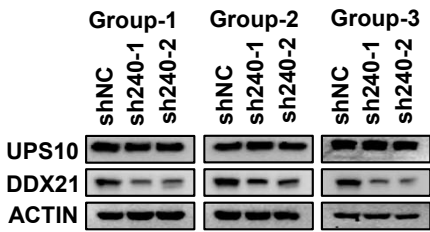

Supplement: Supplementary file 2 — Additional file 2: Supplementary Figure 1. The relative expression levels of LINC00240 in human GES-1, MKN-28, MKN-45, AGS, BGC-823, HGC-27 and MGC-803 cell lines. ***P < 0.001. Supplementary Figure 2. Silencing of LINC00240 significantly promoted apoptosis of gastric cancer cells (A), but did not impact cell cycle (B). Supplementary Figure 3. Expression of apoptotic proteins (A), DDX21 and USP10 (B) in gastric cancer xenografts. [file 13046_2023_2654_MOESM2_ESM.pdf]
